# INTER-ORGANELLE CROSSTALK IN THE REGULATION OF AGING AND LONGEVITY

**DOI:** 10.1093/geroni/igad104.1132

**Published:** 2023-12-21

**Authors:** Meng Wang

## Abstract

In eukaryotic cells, specific functions are compartmentalized into various organelles that are evolutionarily conserved across species. Many of these organelles play crucial roles in regulating cellular homeostasis and organismal health. Lysosomes, mitochondria, lipid droplets, and endoplasmic reticulum (ER) are highly metabolic active and support the metabolic balance of lipids, amino acids, carbohydrates, and nucleotides. These organelles also regulate signal transduction, transcriptional responses, and epigenetic dynamics, and contribute to the control of lifespan and healthspan in a variety of organisms. Importantly, these organelles do not act in isolation, instead exhibit complex intercommunication at structural, metabolic and signaling levels. The dysfunction of these organelles and their intercommunications have been associated with aging and age-related pathologies. This symposium aims to encourage innovative studies on the role of organelle interactions and communications in regulating aging and longevity. Speakers have been invited to share their new findings in this research topic. We will discuss how lysosomal metabolism couples with lysosomal signaling to regulate organelle crosstalk, tissue communication, and organism lifespan; how ER and mitochondria communicate through calcium signaling and promote longevity; how lipid droplets play a novel role in remodeling carbohydrate metabolism and define the metabolic balance in supporting organism health; and how lysosomal activity, mitochondrial function and cytosolic proteostasis orchestrate to support cellular homeostasis.

